# Lithium Treatment Is Safe in Children With Intellectual Disability

**DOI:** 10.3389/fnmol.2018.00425

**Published:** 2018-11-22

**Authors:** Junying Yuan, Juan Song, Dengna Zhu, Erliang Sun, Lei Xia, Xiaoli Zhang, Chao Gao, Galila Agam, Xiaoyang Wang, Klas Blomgren, Changlian Zhu

**Affiliations:** ^1^Henan Key Laboratory of Child Brain Injury, Department of Child Rehabilitation, The Third Affiliated Hospital of Zhengzhou University, Zhengzhou, China; ^2^Henan Key Laboratory of Child Brain Injury, The Third Affiliated Hospital of Zhengzhou University, Zhengzhou, China; ^3^Department of Child Rehabilitation, Children’s Hospital of Zhengzhou University, Zhengzhou, China; ^4^Department of Clinical Biochemistry and Pharmacology and Psychiatry Research Unit, Faculty of Health Sciences, Mental Health Center, Ben-Gurion University of the Negev, Beersheba, Israel; ^5^Perinatal Center, Institute of Neuroscience and Physiology, Sahlgrenska Academy, University of Gothenburg, Gothenburg, Sweden; ^6^Department of Women’s and Children’s Health, Karolinska Institutet, Stockholm, Sweden; ^7^Pediatric Hematology and Oncology, Karolinska University Hospital, Stockholm, Sweden; ^8^Centre for Brain Repair and Rehabilitation, Institute of Neuroscience and Physiology, Sahlgrenska Academy, University of Gothenburg, Gothenburg, Sweden

**Keywords:** intellectual disability, cognition, lithium, safety, children

## Abstract

Lithium is a widely used and effective treatment for individuals with psycho-neurological disorders, and it exhibits protective and regenerative properties in multiple brain injury animal models, but the clinical experience in young children is limited due to potential toxicity. As an interim analysis, this paper reports the safety/tolerability profiles of low-dose lithium treatment in children with intellectual disability (ID) and its possible beneficial effects. In a randomized, single-center clinical trial, 124 children with ID were given either oral lithium carbonate 6 mg/kg twice per day or the same dose of calcium carbonate as a placebo (*n* = 62/group) for 3 months. The safety of low-dose lithium treatment in children, and all the adverse events were monitored. The effects of low-dose lithium on cognition was evaluated by intelligence quotient (IQ), adaptive capacity was assessed by the Infant-Junior Middle School Students Social-Life Abilities Scale (IJMSSSLAS), and overall performance was evaluated according to the Clinical Global Impression-Improvement (CGI-I) scale. After 3 months of lithium treatment, 13/61 children (21.3%) presented with mild side effects, including 4 (6.6%) with gastrointestinal symptoms, 4 (6.6%) with neurological symptoms, 2 (3.3%) with polyuria, and 3 (4.9%) with other symptoms—one with hyperhidrosis, one with alopecia, and one with drooling. Four children in the lithium group had elevated blood thyroid stimulating hormone, which normalized spontaneously after lithium discontinuation. Both IQ and IJMSSSAS scores increased following 3 months of lithium treatment (*F* = 11.03, *p* = 0.002 and *F* = 7.80, *p* = 0.007, respectively), but such increases were not seen in the placebo group. CGI-I scores in the lithium group were 1.25 points lower (better) than in the placebo group (*F* = 82.66, *p* < 0.001) after 3 months of treatment. In summary, lithium treatment for 3 months had only mild and reversible side effects and had positive effects on cognition and overall performance in children with ID.

**Clinical Trial Registration:** Chinese Clinical Trial Registry, ChiCTR-IPR-15007518.

## Introduction

Lithium has been used for decades to treat bipolar disorder, protecting against both depression and mania (Baldessarini et al., 2018). Clinical practice guidelines have long recommended lithium as a first-line long-term treatment for bipolar disorder, but its use has decreased, partly because of safety concerns (Gitlin, 2016). Given the narrow toxicity/therapeutic ratio of this agent in children (McKnight et al., 2012; Pisano et al., 2017), the lack of pharmacotherapeutic guidelines for its use in pediatrics, and the need for routine monitoring of serum concentrations and endocrine and renal function, lithium has been underused in pediatric populations (Grant and Salpekar, 2018). Recently, a double-blind, placebo-controlled study in a pediatric bipolar disorder study showed that lithium was generally well tolerated and that the adverse effects were acceptable for most participants (Findling et al., 2015). Furthermore, a clinical study with fragile X syndrome showed that both children and young adults could benefit from lithium treatment and that the side effects were well tolerated (Berry-Kravis et al., 2008). There are indications that lithium might be a treatment option for children with mood disorders in general and bipolar disorder in particular, as well as for intellectual disorders (Campbell et al., 1995; Berry-Kravis et al., 2008; McKnight et al., 2012; Aprahamian et al., 2014; Liu and Smith, 2014; Siegel et al., 2014).

Intellectual disability (ID) is characterized by significant limitations both in intellectual functioning and in adaptive behavior, as expressed in conceptual, social, and practical adaptive skills, and it is most often apparent before the age of 18 (Schalock et al., 2007; Brosco et al., 2013). The prevalence of ID is estimated to be 1–3% (van Bakel et al., 2014; Van Naarden Braun et al., 2015; Maenner et al., 2016). Rehabilitation and education are currently the main therapeutic interventions employed for children with ID, but their effects are limited (Sturmey, 2012; Picker and Walsh, 2013). As knowledge of the underlying neurochemical pathways of these deficits has improved, studies of targeted drugs such as lithium that alleviate cognitive deficits have been reported in animal models, such as models of Down syndrome (Contestabile et al., 2013). Lithium treatment has been shown to be neuroprotective in multiple *in vitro* cell culture systems, including hippocampal neural stem/progenitor cells (Zanni et al., 2015), cerebellar granular cells (Wei et al., 2000), and cerebral cortical cells (De-Paula et al., 2016). It has also been found to protect against brain damage induced by cerebral irradiation (Huo et al., 2012; Zhou et al., 2017), hypoxia-ischemia (Li et al., 2010, 2011; Xie et al., 2014), and trauma (Shim and Stutzmann, 2016) in animal models and to improve synaptic plasticity, neurogenesis, and memory in a mouse model of Down syndrome (Contestabile et al., 2013; Guidi et al., 2017).

The following reports indicate that lithium treatment might have beneficial effects on cognitive performance: lithium treatment reversed cognitive deficits in a mouse model of fragile X syndrome (King and Jope, 2013) and in individuals with fragile X syndrome, Alzheimer’s disease and mild cognitive impairment (Berry-Kravis et al., 2008; Matsunaga et al., 2015). Given that potential toxicity of lithium is dose-dependent and reversible, and because lithium has beneficial potential for children with behavior problems and mood/intellectual disorders, our hypothesis was that low-dose lithium treatment for children with ID is safe and can improve cognitive performance.

## METHODS

### Study Population

This study was a single center, double-blinded, randomized control trial. Participants were recruited from the Child Rehabilitation Center of the Third Affiliated Hospital of Zhengzhou University between March 2016 and June 2017. Informed and written parental consent was acquired from all individual participants included in the study according to the World Medical Association’s Declaration of Helsinki. The study procedures and the protocol were approved by the Human Research Ethics Committee of the Third Affiliated Hospital of Zhengzhou University (2015/AFZZ/15), and the study was registered in the Chinese Clinical Trial Registry of International Clinical Trials Registry Platform under the World Health Organization/International Clinical Trials Registry Platform (ChiCTR-IPR-15007518). However, due to lithium’s potential toxicity, treatment in children has raised much concern. Therefore, an interim analysis was carried out, and the current report’s focus is on safety/tolerability profiles of lithium treatment in children.

A total of 181 children aged 4–11 years with suspected ID were deemed eligible for the study. All of the participants were further evaluated by a pediatric psychiatrist and by chromosome analysis. Inclusion criteria were ① diagnostic criteria for ID, meaning an intelligence quotient (IQ) <70 as evaluated by the Wechsler Intelligence Scale for Children-Fourth Edition, China Revised (WISC-IV, CR) or the Wechsler Preschool and Primary Scale of Intelligence-Fourth Edition, China Revised (WPPSI-IV, CR), and evidence of deficits or impairments in adaptive skills, and ② an available parent for all clinical assessments and examinations and at least one parent having acceptable reading skills. Exclusion criteria were ① inherited metabolic disorders, ② confirmed chromosomal abnormalities, ③ medication that might affect cognitive performance or cause damage to vital organs, ④ kidney disease, ⑤ thyroid disease, ⑥ suspected autism spectrum disorder, ⑦ mania or depression.

### Study Design and Treatment Procedures

The 124 participants who fulfilled the inclusion criteria were numbered according to enrollment sequence. The study medication [lithium carbonate (treatment group) or calcium carbonate (placebo group)] was randomly assigned in a 1:1 allocation to each individual number in advance using a computer-based random-number generator where the fixed random seed was set to March 2016. In the lithium carbonate treatment group, 62 participants were given lithium carbonate tablets at 6 mg/kg twice per day (with a 12 h interval) for 3 months, and this dosage is well tolerated according to a previous study for fragile X syndrome with lithium treatment for 2 months and provides functional benefits (Berry-Kravis et al., 2008). In the placebo group, 62 children were given 6 mg/kg calcium carbonate. Serum lithium levels were measured after 1, 2, 4, 8, and 12 weeks of treatment. No concomitant drugs and no rehabilitation measures were given during the study period in either group. Discontinuation was defined as ① children who were lost to follow up, ② children who did not complete the treatment, or ③ children who experienced serious side effects. Criteria for withholding or stopping the study included abnormal kidney or thyroid function as indicated by blood tests and grade 3 or greater side effects according to the Common Terminology Criteria for Adverse Events, version 4.0.

### Cognitive Assessment

#### Intelligence Quotient

The WISC-IV, CR and the WPPSI-IV, CR are widely accepted and used to assess IQ in children of different ages, and the lowest possible score is 40 (Molinero et al., 2015). All children in the study ≥6 years old were tested using the WISC-IV, CR, and children <6 years old were tested using the WPPSI-IV, CR in this study.

#### Adaptive Behavior

The Infant-Junior Middle School Students Social-Life Abilities Scale (IJMSSSLAS) is an adaptive behavioral scale including the following six items: self-help, locomotion, occupation, communication, socialization, and self-direction. There are 132 items in this scale, and the child gets one point for each item for a total possible score of 132. The raw scores can be transformed into a standard score that is adjusted for age. The standard score is divided into six grades: a standard score ≥10 is considered normal, 9 is a borderline level, 8 is a mildly borderline level, 7 is medium abnormal, 6 is severely abnormal, and 5 is profoundly abnormal (Liu et al., 2010). The scale is used to assess the adaptiveness of children aged 0–17 years old.

#### Clinical Global Impression-Improvement (CGI-I)

Clinical Global Impression-Improvement is a single item that rates changes in overall clinical manifestation from 1 to 7 (1 = very much improved global clinical manifestation over the course of treatment; 2 = much improved; 3 = minimally improved; 4 = no change; 5 = minimally worse; 6 = much worse; and 7 = very much worse) (Bailey et al., 2016). At the end of 3-month treatment period, the clinicians rated the child’s change of overall clinical manifestation, and this was referenced to the scores given by parents or guardians according to the CGI-I scale. The clinicians who performed the evaluation of the CGI-I score were blinded to group allocation of the children and were not allowed to inquire their treatment history.

These parameters were evaluated by a certified clinician blinded to the treatment. WISC-IV, CR, WPPSI-IV, CR, and IJMSSSLAS were evaluated pre-treatment and after 3 months of treatment in all children in both groups. All children underwent CGI-I evaluation after 3 months of treatment.

### Monitoring Safety and Adverse Events

Every individual in both groups and his/her guardian were given an adverse event monitoring checklist that included the following common clinical side effects: decreased appetite, polydipsia, polyuria, vomiting, nausea, tiredness, drowsiness, tremor, dullness, hyperactivity, seizures, aggressive behavior, and drooling. The parents/guardians recorded the time of occurrence and severity of the symptom and were asked to fill out the form whenever any symptom or discomfort occurred and to bring the form back when the child was re-examined after 3 months. All participants and their guardians were encouraged to call the clinician in case of perceived side effects, and the clinician called all of the children’s guardians to inquire about the children once a month. All participants and their guardians were instructed that if the symptom of a suspected side effect was tolerated and transient, it should be recorded as “mild”; if the symptom of a suspected side effect was not tolerated and lasted for 2 days, they were to report this to the clinician as soon as possible.

Laboratory examinations, including liver function (alanine transaminase and aspartate transaminase), renal function (blood urea nitrogen, serum creatinine, and uric acid), blood cell counts, and thyroid function [thyroid stimulating hormone (TSH), free T3 and free T4], were performed every 4 weeks during the treatment. If abnormal laboratory values were found, the participants were informed and re-examined after 2 weeks.

### Monitoring Serum Lithium Concentrations

Oral lithium or calcium carbonate was started when the screening procedures were completed. Serum lithium levels were measured at 1, 2, 4, 8, and 12 weeks of treatment using an ion-selective electrode method. Blood was withdrawn at 2 h and 12 h after the morning administration.

### Drop-Outs

For individuals who dropped out of the intervention, a follow-up assessment was planned and, if possible, the reasons for dropping out were recorded. Intention-to-treat analyses were conducted.

### Statistical Analysis

Data were analyzed using SPSS 17.0 (IBM, Armonk, NY). Quantitative data are expressed as means ± SD. Gender and rates of abnormal brain MRI were compared by chi-square test, and intra- and inter-group data from laboratory examinations, IQ, LJMSSSLAS, and IGC-I were analyzed by independent *t*-test or repeated-measures ANOVA. The level of statistical significance was set at two-tailed a = 0.05 or adjusted a = 0.025 (when run repeated measurement ANOVA).

## Results

### Participant Characteristics

**FIGURE 1:**
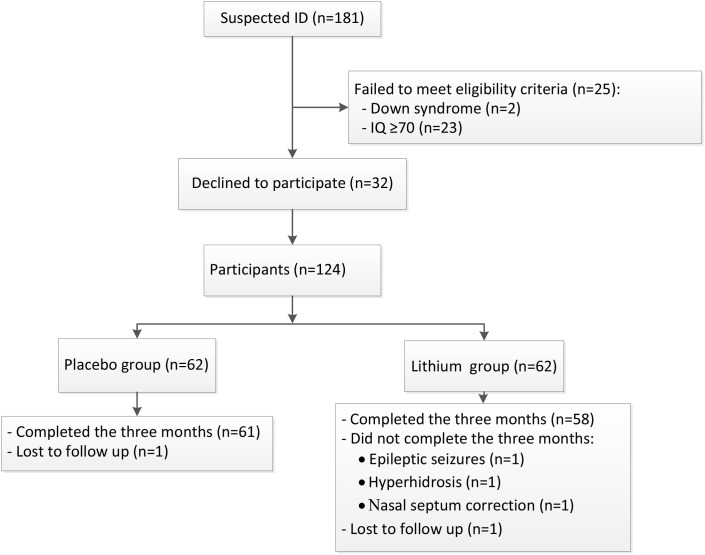
The study flow. A schematic flowchart shows the number of participants who were screened for eligibility, randomly assigned to the lithium or placebo groups, and followed up to 3 months. Lost to follow-up means that contact with the family was lost during the follow-up period.

Of 181 children with suspected ID, 23 children with an IQ ≥ 70 and 2 with confirmed Down syndrome were excluded. Of the 156 remaining eligible children, 32 declined participation, and finally 124 children were enrolled in the study (Figure 1). The mean age of the lithium group was 74.8 ± 21.8 months (range 48–132 months) and that of the placebo group was 82.2 ± 25.6 months (range 48–133 months). The lithium and the placebo groups were matched for age, gender, IQ score, IJMSSLAS score, and CGI-I score (Table 1). One child in each group was lost to follow-up, and three children in the lithium group did not finish the 3 months of treatment, one due to epileptic seizures, one due to hyperhidrosis, and one due to surgery to correct nasoseptal deviation (Figure 1).

**Table 1:** Demographic data.

Parameter	Placebo group (*n* = 62)	Lithium group (*n* = 62)	Difference
Age (months, mean ± SD)	82.2 ± 25.6	74.8 ± 21.8	*t* = 1.739, *p* = 0.085
Boys n/N (%)	41/62 (64.5%)	44/62 (66.1%)	χ^2^ = 0.337, *p* = 0.562
Cerebral palsy n/N (%)	8/62 (14.5%)	14/62 (21.0%)	χ^2^ = 1.989, *p* = 0.158
Abnormal brain MRI^a^ n/N (%)	20/42 (47.6%)	12/36 (33.3%)	χ^2^ = 1.635, *p* = 0.201

*^*a*^Abnormal brain MRI indicates that a structural anomaly was seen by MRI, including congenital aplasia and acquired changes after injury such as periventricular leukomalacia*.

### Clinical Symptoms

The adverse events observed during lithium treatment are summarized in Table 2. No serious adverse events (grade 3 and above) were reported, but 13/61 children (21.3%) had mild symptoms. Of these, 4/61 children (6.6%) presented with gastrointestinal symptoms (including nausea, vomiting and decreased appetite), 4/61 (6.6%) had neurological symptoms, and 2/61 (3.3%) had polyuria—all of which were regarded as lithium-induced side effects. An additional 3/61 (4.9%) children had other symptoms, including one with hyperhidrosis, one with alopecia, and one with drooling. Surgery to correct nasoseptal deviation in the child mentioned above was assumed to be unrelated to lithium therapy (Table 2). Although the adverse events were transient and mild, three children and their guardians decided to withdraw their consent and to drop out of the study. Thus, 58 lithium-treated children completed 3 months of treatment. In the placebo group, two children were reported to suffer from nausea and appetite loss (Table 2).

**Table 2:** Adverse events during the treatment period.

	Placebo group, *n* = 61		Lithium group, *n* = 61	
	*n*	%	*n*	%
**Urinary symptoms**	0	0	2	3.28%
Polyuria	0		2	
**Gastrointestinal symptoms**	2	3.28%	4	6.56%
Nausea	1		2	
Vomiting	0		1	
Decreased appetite	1		1	
**Neurological symptoms**	1	1.64%	4	6.56%
Aggressive behavior	0		1	
Epileptic seizures	1		1	
Dullness	0		1	
Hyperactivity	0		1	
**Other symptoms**	0	0	4	6.56%
Alopecia	0		1	
Drooling	0		1	
Hyperhidrosis	0		1	
Nasoseptal surgery	0		1	
**Total**	3	4.92%	14	21.31%^a^

*^*a*^The 14 total events included 1 “nasoseptal surgery,” which was regarded as unrelated to lithium administration, so the total number of children who presented with side effects was 13 when the percentages were calculated*.

### Laboratory Examinations

Serum lithium levels of the 58 children who completed 3 months of lithium treatment were 0.47–0.92 mmol/L at 2 h after the morning dose and 0.09–0.46 mmol/L 12 h after the morning dose, just before the evening dose.

Liver function, kidney function, and blood cell counts showed no clinically significant changes during the treatment period in either the lithium or placebo groups. Thyroid function, as indicated by free T3, free T4, and TSH, did not show significant differences in the lithium treatment group compared to the placebo treatment group; however, elevated TSH blood levels were found in four children at the end of the 3 months of treatment in the lithium group, but all of them normalized spontaneously within 2–8 weeks after discontinuation of the treatment. There was no significant change in electrocardiograms in any of the children after 3 months of treatment (Table 3).

**Table 3:** Laboratory examinations during the treatment period (means ± SD).

	Placebo group		Lithium group	
	Pre-treatment	Post-treatment	Pre-treatment	Post-treatment
**Liver function**				
Alanine transaminase (U/l)	16.9 ± 8.9	17.8 ± 7.3	18.5 ± 11.1	17.8 ± 9.9
Aspartate transaminase (U/l)	26.4 ± 4.3	27.2 ± 8.6	29.8 ± 11.3	25.8 ± 5.4
γ-glutamyltransferase (U/l)	12.8 ± 3.4	12.5 ± 3.3	13.1 ± 5.2	16.8 ± 12.74
Alkaline phosphatase (U/l)	243.1 ± 53.4	218.0 ± 70.6	221.2 ± 61.7	214.6 ± 68.4
Total protein (g/l)	68.7 ± 3.6	67.8 ± 3.1	69.1 ± 4.3	68.9 ± 4.6
Albumin (g/l)	46.4 ± 2.7	46.8 ± 3.9	46.4 ± 2.9	47.2 ± 3.3
Globulin (g/l)	22.3 ± 3.1	21.0 ± 3.2	22.7 ± 3.2	21.7 ± 2.7
Total bilirubin (μmol/L)	9.4 ± 2.6	9.3 ± 3.7	9.5 ± 3.8	9.2 ± 2.6
Direct bilirubin (μmol/L)	2.0 ± 0.8	2.3 ± 0.8	2.3 ± 1.1	2.2 ± 0.7
Indirect bilirubin (μmol/L)	7.5 ± 2.4	7.0 ± 2.9	7.8 ± 4.6	7.0 ± 2.1
**Renal function**				
Urea (mmol/L)	4.5 ± 1.0	3.5 ± 0.5	4.6 ± 1.2	4.4 ± 1.3
Creatinine (μmol/L)	31.7 ± 6.9	29.2 ± 3.8	31.0 ± 7.5	33.0 ± 7.7
Uric acid (μmol/L)	228.9 ± 83.2	241.0 ± 103.6	242.0 ± 75.6	259.6 ± 74.6
**Thyroid function**				
Free T3 (pmol/L)	5.8 ± 0.5	5.8 ± 0.4	5.8 ± 0.8	5.9 ± 0.5
Free T4 (pmol/L)	15.1 ± 2.7	15.1 ± 2.5	15.6 ± 2.2	16.3 ± 1.9
Thyroid stimulating hormone (mIU/L)	2.8 ± 1.9	2.6 ± 1.6	2.8 ± 1.6	3.4 ± 1.7
**Routine blood tests**				
White blood cells (10^9^/L)	6.7 ± 1.5	8.8 ± 1.5	7.9 ± 3.6	7.6 ± 3.2
Red blood cells (10^12^/L)	4.6 ± 0.4	4.7 ± 0.4	4.4 ± 0.5	4.6 ± 0.4
Hemoglobin (g/L)	125.3 ± 8.8	129.5 ± 13.4	122.0 ± 10.7	124.1 ± 12.6
Platelets (10^9^/L)	186.9 ± 19.8	190.0 ± 21.6	189.4 ± 25.8	193.5 ± 29.9

*There were no significant differences for any of the parameters between the two groups before and after treatment*.

### IQ and Adaptive Behavior

Although there were no significant differences in either IQ or IJMSSSLAS scores between the placebo and the lithium groups after 3 months of treatment, paired comparisons with each group indicated that IQ (*F* = 11.03, *p* = 0.002) and IJMSSSLAS scores (*F* = 7.80, *p* = 0.007) in the lithium group (but not in the placebo group) were significantly increased. In addition, lithium treatment significantly decreased the CGI-I scores (*F* = 82.66, *p* < 0.001) (Table 4).

**Table 4:** Comparisons for IQ, IJMSSSLAS and IGI-I.

		Placebo group	Lithium group
IQ	Baseline	47.35 ± 9.30	45.90 ± 9.16
	Post-treatment	46.59 ± 10.12	46.87 ± 10.76a
IJMSSSLAS	Baseline	6.42 ± 1.95	6.26 ± 1.29
	Post-treatment	6.36 ± 1.20	6.51 ± 1.42a
CGI-I	Post-treatment	3.60 ± 0.49	2.37 ± 0.93b

*The IQ, IJMSSSLAS, and CGI-I scores are shown as means ± SD. Data were analyzed by repeated-measures ANOVA. ^*a*^Compared post-treatment with baseline, *p* < 0.01. ^*b*^Compared lithium group with placebo group, *p* < 0.001*.

## Discussion

The results presented here are an interim analysis aimed at assessing the safety/tolerability profiles and possible beneficial effects of lithium treatment in 4- to 11-years-old children with ID. To the best of our knowledge, this is the first report of a randomized, double-blind, placebo-controlled clinical evaluation the safety of low-dose lithium as a potential therapeutic agent in children with ID.

We intentionally kept the dosage low (6 mg/kg lithium carbonate twice per day resulting in lithium blood levels of 0.4–0.92 mmol/L at 2 h after the morning dose and 0.09–0.46 mmol/L 12 h after the morning dose, just before the evening dose) to minimize the risk of adverse events and poor compliance. Indeed, all adverse events were transient and mild, and most individuals could tolerate the treatment well and completed the 3 months of treatment. Of 61 children on lithium, 14 were found to have mild adverse events, 13 of which were well-known lithium-induced side effects. This prevalence (about 21.3%) is considerably lower than that in Berry-Kravis and colleague’s study (Berry-Kravis et al., 2008) in which about half of the 15 individuals with fragile X syndrome presented with lithium-related side effects after lithium (20 mg/kg/day) was administered three times a day titrated to achieve serum levels of 0.8–1.2 mmol/L. The greater prevalence of side effects in their study was apparently attributable to the higher dose, as has also been implied from other studies (Silva et al., 1992; Masi et al., 2009).

It has been widely accepted that lithium-induced side effects are dose-dependent (Malhi and Berk, 2012; McKnight et al., 2012). Because the therapeutic window of lithium is relatively narrow in the treatment of bipolar disorders (Haussmann et al., 2015), higher lithium concentrations are often necessary for maintaining the therapeutic effect in individuals with bipolar disorder. Recently, more novel biological properties of lithium have been emphasized, supporting it as a candidate drug for the prevention and treatment of brain injury and cognitive impairment (Li et al., 2011; Huo et al., 2012; Zhou et al., 2017). Lithium has been shown to be effective for such purposes at lower concentrations, which helps to avoid potential toxicity, and low-dose lithium treatment is tolerable and safe for long-term treatment even in elderly patients (Aprahamian et al., 2014). Another study compared different lithium treatment regimens and found that once-daily administration appears to be less toxic than multiple daily dose regimens (Grandjean and Aubry, 2009). In the current study, low-dose lithium (6 mg/kg, twice per day) in children was well tolerated and beneficial effects were observed in children with ID. This indicates that the current treatment regimen is acceptable, but further optimization of the therapeutic protocol is needed based on studies with larger populations.

Gastrointestinal symptoms (including nausea, vomiting, and decreased appetite) accounted for approximately 30% of all of adverse events, and it is possible that the frequency of gastrointestinal side effects could be reduced by using lithium citrate instead of lithium carbonate (Vasile and Shelton, 1982). The hyperhidrosis experienced by one child in the lithium treatment group might have been related to the treatment because it subsided 2 weeks after lithium discontinuation, but the mechanism behind such a side effect remains unclear. Epileptic seizures experienced by one child in the lithium treatment group were not likely related to lithium because even though this is debated lithium has also been shown to prevent seizures (Shukla et al., 1988); furthermore, there was also one child with seizures in the placebo group.

Elevated blood TSH is a known dose-dependent and reversible side effect of lithium treatment (Berry-Kravis et al., 2008). However, the present observation of elevated TSH levels in 6.6% of the individuals in the lithium group, with normal free T3 and T4 is less frequent than lithium-induced thyroid abnormalities previously described in the literature (Berry-Kravis et al., 2008; Sethy and Sinha, 2016). Moreover, TSH levels in the present study normalized within 2–8 weeks after lithium withdrawal in all afflicted children.

Kidney function impairment in individuals taking lithium was first reported as a possible side effect in the 1970s (Lee et al., 1971). Among the renal function abnormalities reported, lithium induces renal tubular dysfunction that is often clinically manifested as cumulative, dose-related, and reversible (Clos et al., 2015; Gong et al., 2016). Consistent with Aprahamian et al. (2014), who reported no impairment in renal function in low-dose lithium-treated elderly individuals, only 3.3% of the children in the lithium group in the present study suffered from polyuria, and none of these children had kidney function impairment. It might therefore be concluded that at the dosage used lithium was safe in children aged 4–11 years. However, it must be noted that there were only 58 children who completed the oral administration of lithium carbonate for 3 months, and more children and longer treatment durations might be needed to evaluate the side effects of lithium carbonate more thoroughly.

To further evaluate whether lithium treatment affected ID, we used scales that correlate with IQ (Berry-Kravis et al., 2008; Hassiotis et al., 2011; Flatt-Fultz and Phillips, 2012). The average IQ did not differ between the lithium and the placebo groups after 3 months of treatment, but unlike in the placebo group the average IQ did increase significantly in children in the lithium group compared with the average IQ before the treatment. Interestingly, a Danish nationwide, population-based, nested case–control study of 73,731 patients with dementia and 733,653 control individuals found that the level of lithium exposure in the drinking water was lower for patients with a diagnosis of dementia than for controls. Furthermore, compared with individuals exposed to 2.0–5.0 μg/L, the incidence rate ratio of dementia was significantly decreased in those exposed to more than 15.0 μg/L (Kessing et al., 2017). Although the Danish study relates to adults and to lithium in the drinking water (a dose several fold lower than when given as medication), it corroborates with our results even though there was only a mild increase in IQ points in the treatment group. It remains to be tested whether a higher dose and/or a longer treatment period would have a stronger effect on cognitive performance and whether the effect of lithium is long-lasting. Given that in both groups the majority of children whose IQ values did not change had IQ ≤ 40, and due to the floor effect in the Wechsler intelligence test, alternative tools would be required to detect moderate changes in cognitive performance in general and in those with low IQ in particular.

The current study has some limitations. First, the number of participants was not large enough for a full safety evaluation, and more participants and more age groups as well as longer treatment and follow up times points are needed to detect infrequent safety issues. Second, ID is a neurodevelopmental disorder with multiple etiologies. The participants with confirmed chromosome abnormalities, congenital structural, and functional abnormalities, and infection or trauma-related ID were excluded, and only children with ID with unknown etiology were included in the study. This was done to make the treatment group and placebo group comparable and to reduce the influence of different etiologies on any therapeutic effects. However, it would be valuable to study lithium treatment in ID with specific etiologies, such as fragile X syndrome and Down syndrome. Third, 3 months was a short period for evaluating the therapeutic effect in children with ID, and learning effects should be considered. Even though WISC-IV and WPPSI-IV are useful for measuring changes in IQ, the sensitivity of the instruments was not high enough for children with IQ ≤ 40. Fourth, as a landmark drug for manic-depressive psychosis lithium has been used to treat behavior disorder in children even when they are too young to get the diagnosis of bipolar disorder, and lithium has been shown to improve behavior in children (Findling et al., 2015), but behavioral issues were not explored in the present study.

In summary, low-dose lithium treatment was well tolerated and improved cognitive performance and adaptive behavior in children with ID without causing severe or irreversible side effects. These preliminary results encourage further studies to be carried out for more extended periods using better evaluation tools of cognitive performance in children with profound ID in order to assess the long-term effects on cognitive performance and to explore the safety, efficacy, and generalizability of lithium treatment.

## Ethics Statement

Informed parental consent was acquired from all individual participants included in the study according to the World Medical Association’s Declaration of Helsinki. The study procedures and the protocol were approved by the Human Research Ethics Committee of the Third Affiliated Hospital of Zhengzhou University (2015/AFZZ/15), and the study was registered with the Chinese Clinical Trial Registry of International Clinical Trials Registry Platform in World Health Organization (ChiCTR-IPR-15007518).

## Author Contributions

JY, JS, and DZ developed and led the intervention, designed the data collection, conducted the statistical analyses, and drafted the initial manuscript. ES performed the data collection. LX, XZ, and CG performed the intervention and data collection. GA participated in drafting and finalizing the manuscript. XW and KB conceptualized and designed the study and participated in drafting and finalizing the manuscript. CZ conceptualized and designed the study, developed the intervention, designed the data collection, and participated in drafting and finalizing the manuscript.

## Conflict of Interest Statement

The authors declare that the research was conducted in the absence of any commercial or financial relationships that could be construed as a potential conflict of interest.
